# Supplementary material for: Modulation of the Tumor Microenvironment with Trastuzumab Enables Radiosensitization in HER2+ Breast Cancer
Source: Cancers (Basel). 2022 Feb 17;14(4):1015. doi: 10.3390/cancers14041015 (PMC8869800; doi:10.3390/cancers14041015)
Supplement: Supplementary file 1 [file cancers-14-01015-s001.zip › Figure S2.pdf]

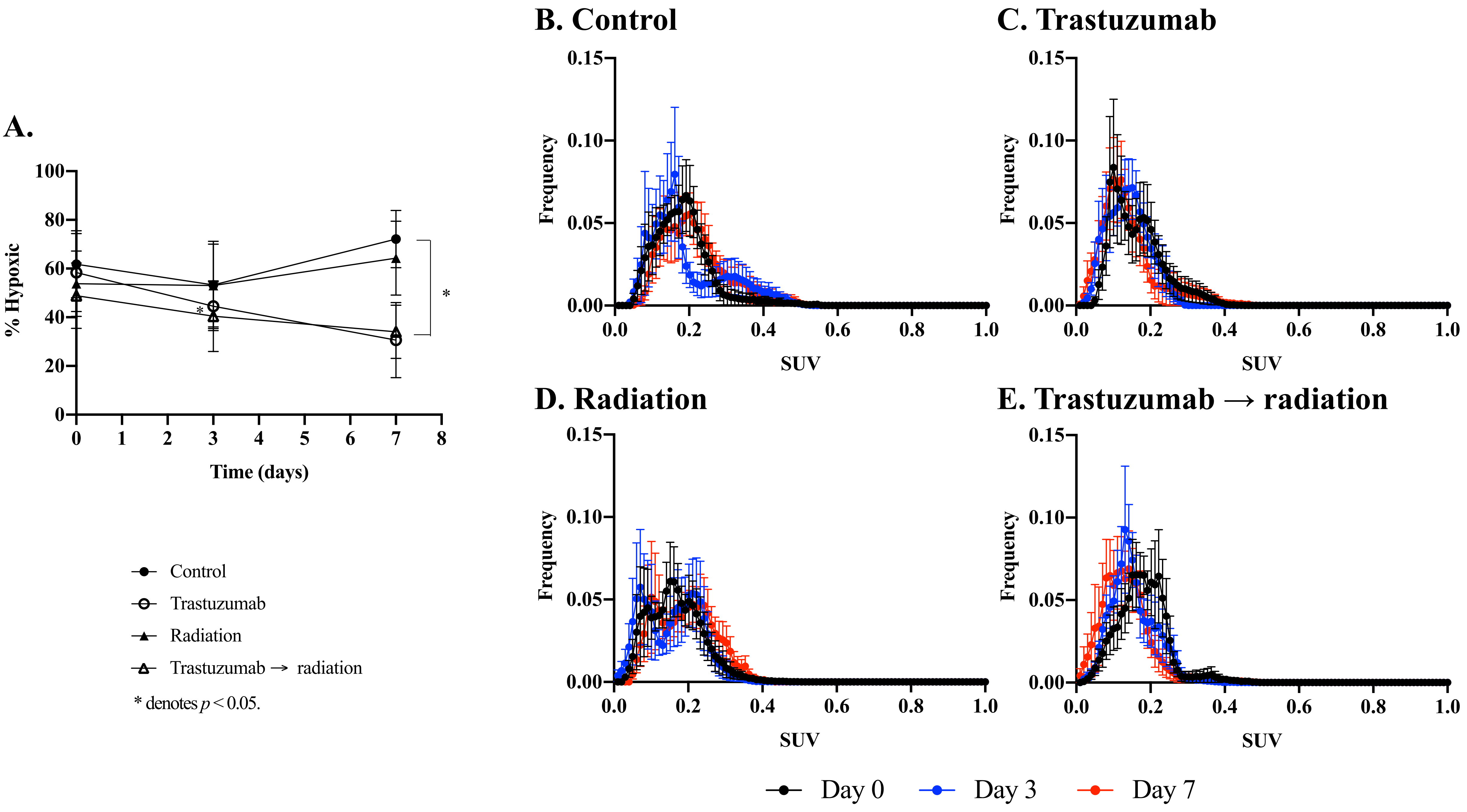
A.
% Hypoxic
Time (days)
*
Control
Trastuzumab
Radiation
Trastuzumab → radiation
* denotes p < 0.05.
B. Control
C. Trastuzumab
D. Radiation
E. Trastuzumab → radiation
Frequency
SUV
Day 0
Day 3
Day 7
