# Supplementary material for: Modulation of the Tumor Microenvironment with Trastuzumab Enables Radiosensitization in HER2+ Breast Cancer
Source: Cancers (Basel). 2022 Feb 17;14(4):1015. doi: 10.3390/cancers14041015 (PMC8869800; doi:10.3390/cancers14041015)
Supplement: Supplementary file 1 [file cancers-14-01015-s001.zip › Table S1.pdf]

**TUMOR MODEL**		SUV_{mean}		
**BT474**	Tissue	Day 0	Day 3	Day 7
Control (N = 5)	Tumor	0.19 ± 0.06	0.22 ± 0.10	0.23 ± 0.12
	Muscle	0.11 ± 0.03	0.13 ± 0.04	0.12 ± 0.01
Trastuzumab (N = 6)	Tumor	0.18 ± 0.07	0.13 ± 0.05	0.14 ± 0.08
	Muscle	0.10 ± 0.04	0.13 ± 0.04	0.12 ± 0.01
Fractionated radiation (N = 6)	Tumor	0.16 ± 0.06	0.16 ± 0.08	0.20 ± 0.09
	Muscle	0.12 ± 0.06	0.11 ± 0.05	0.13 ± 0.08
Trastuzumab → Fractionated radiation (N = 7)	Tumor	0.17 ± 0.06	0.15 ± 0.08	0.15 ± 0.06
	Muscle	0.11 ± 0.05	0.09 ± 0.04	0.10 ± 0.03
**MDA-MB-361**	Tissue	Day 0	Day 3	Day 7
Control (N = 5)	Tumor	0.20 ± 0.05	0.19 ± 0.08	0.32 ± 0.10
	Muscle	0.12 ± 0.03	0.11 ± 0.02	0.16 ± 0.05
Trastuzumab (N = 4)	Tumor	0.19 ± 0.01	0.15 ± 0.03	0.18 ± 0.02
	Muscle	0.13 ± 0.01	0.11 ± 0.01	0.14 ± 0.02
Fractionated radiation (N = 5)	Tumor	0.22 ± 0.02	0.23 ± 0.07	0.31 ± 0.06
	Muscle	0.12 ± 0.02	0.11 ± 0.03	0.17 ± 0.10
Trastuzumab → Fractionated radiation (N = 5)	Tumor	0.20 ± 0.03	0.20 ± 0.07	0.19 ± 0.09
	Muscle	0.13 ± 0.03	0.12 ± 0.03	0.15 ± 0.04
**BCM 3472**	Tissue	Day 0	Day 3	Day 7
Control (N = 5)	Tumor	0.13 ± 0.06	0.12 ± 0.04	0.20 ± 0.04
	Muscle	0.09 ± 0.04	0.10 ± 0.04	0.10 ± 0.02
Trastuzumab (N = 5)	Tumor	0.13 ± 0.04	0.15 ± 0.03	0.18 ± 0.10
	Muscle	0.09 ± 0.04	0.10 ± 0.04	0.09 ± 0.02
Fractionated radiation (N = 5)	Tumor	0.12 ± 0.04	0.15 ± 0.04	0.23 ± 0.03
	Muscle	0.10 ± 0.04	0.11 ± 0.04	0.13 ± 0.04
Trastuzumab → Fractionated radiation (N = 6)	Tumor	0.15 ± 0.04	0.19 ± 0.04	0.23 ± 0.05
	Muscle	0.09 ± 0.04	0.12 ± 0.02	0.12 ± 0.02
