# Supplementary figures and images for: Modulation of the Tumor Microenvironment with Trastuzumab Enables Radiosensitization in HER2+ Breast Cancer
Source: Cancers (Basel). 2022 Feb 17;14(4):1015. doi: 10.3390/cancers14041015 (PMC8869800; doi:10.3390/cancers14041015)

**A.**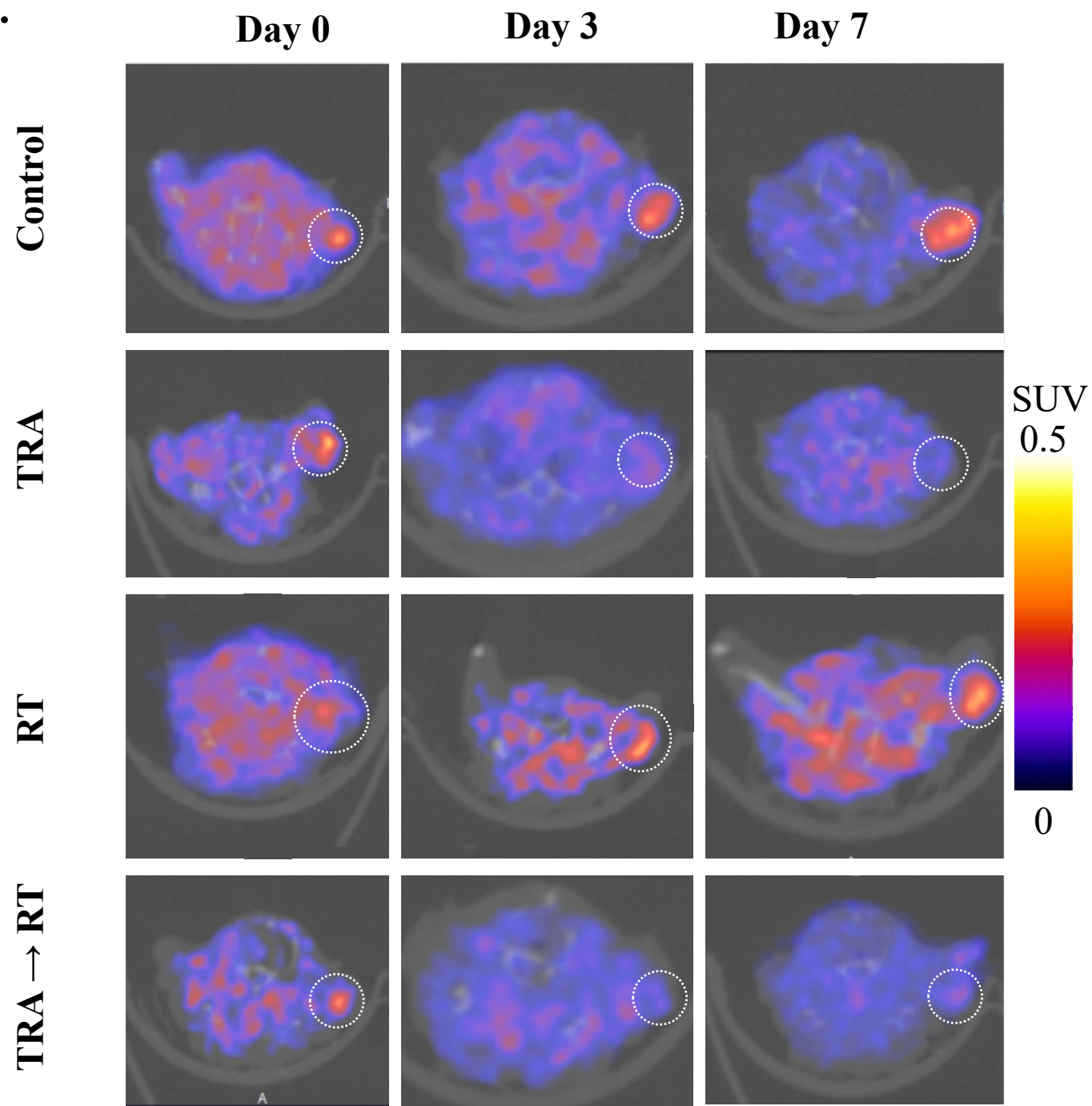**B.**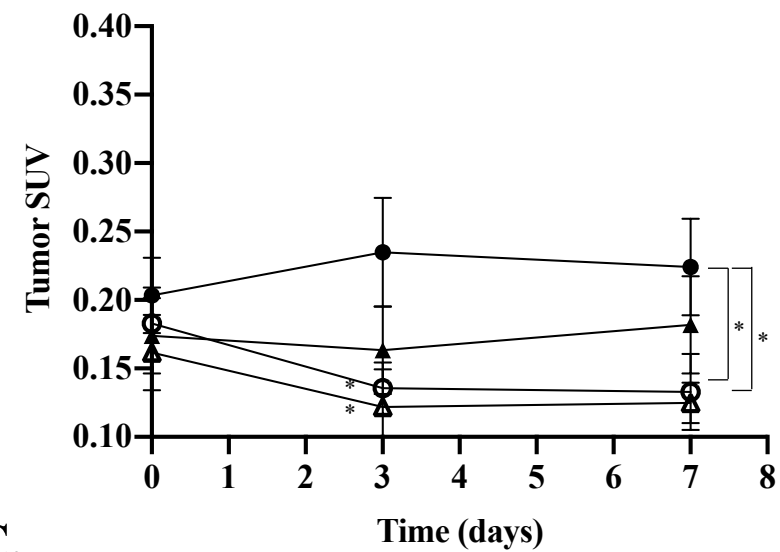**C.**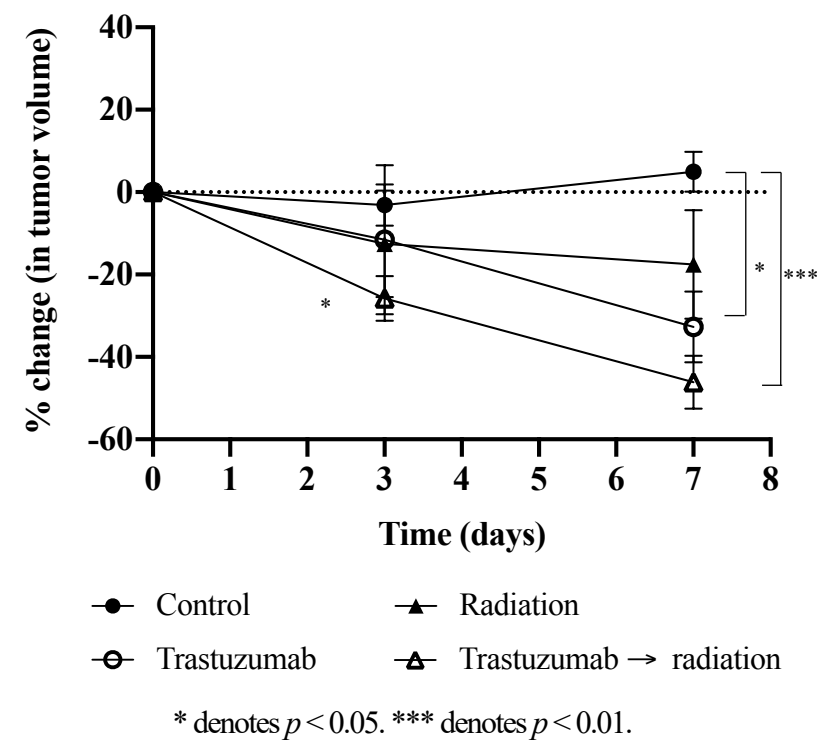

Supplement: Supplementary file 1 [file cancers-14-01015-s001.zip › Figure S1.pdf]

A.

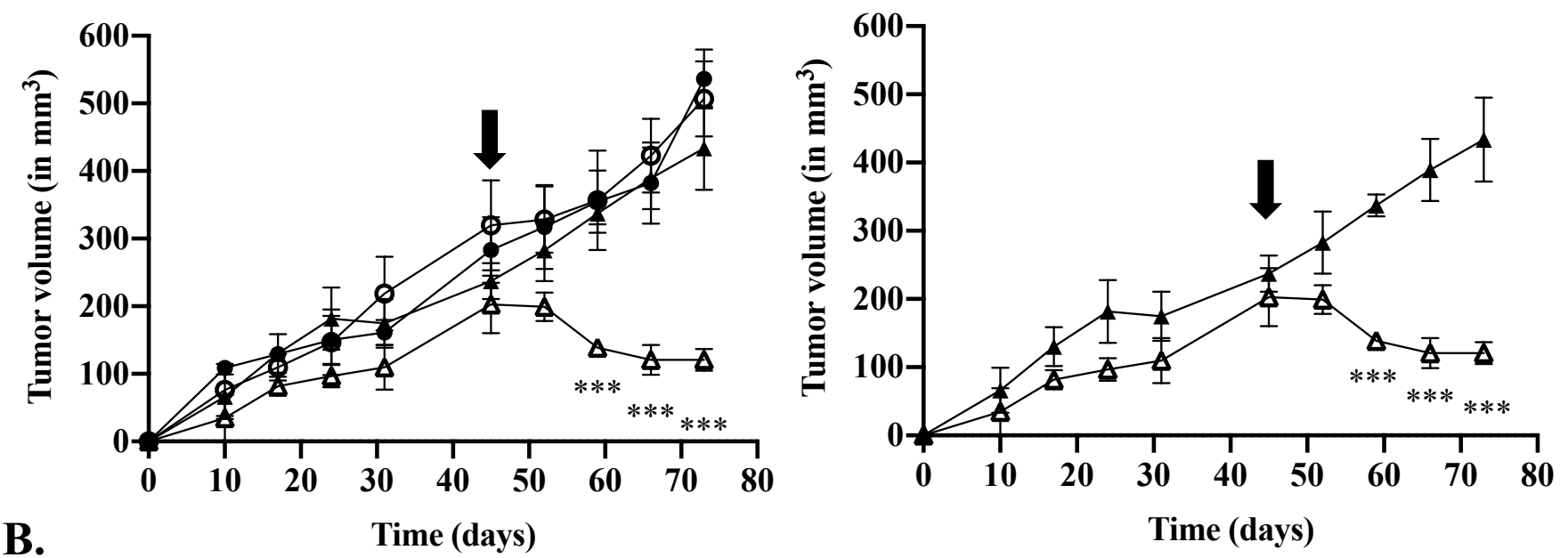

B.

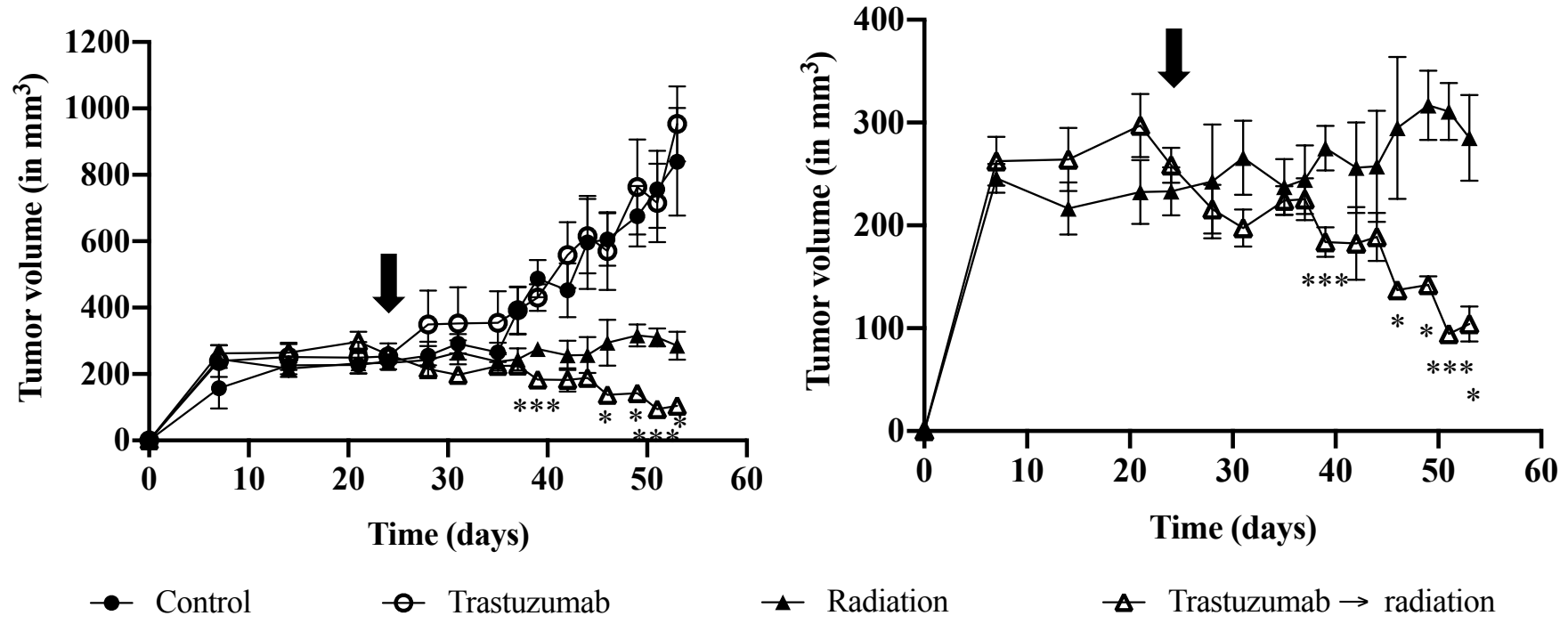

\* denotes  $p < 0.05$ . \*\*\* denotes  $p < 0.01$ .

Supplement: Supplementary file 1 [file cancers-14-01015-s001.zip › Figure S3.pdf]
